# Lone Hepatocellular Carcinoma: An Isolated Chest Wall Malignancy

**DOI:** 10.1155/2017/3531823

**Published:** 2017-08-30

**Authors:** Joseph Allencherril, Sebastian Bruera, Ronan Allencherril, Richard J. Hamill

**Affiliations:** ^1^Department of Medicine, Baylor College of Medicine, One Baylor Plaza, Houston, TX, USA; ^2^University of Texas Medical Branch, Galveston, TX, USA

## Abstract

Herein we describe the case of an elderly diabetic gentleman presenting with a two-week history of dyspnea and nonproductive cough, found to have a large left anterolateral chest wall mass. Further characterization through computed tomography (CT) of the chest revealed a soft tissue mass in the left anterior lower hemithorax found to be hepatocellular carcinoma (HCC). The liver, spleen, and pancreas were unremarkable. Diagnostic labs were unremarkable. The patient had no history of hepatitis, alcohol abuse, or illicit substance use. Pathological examination and immunohistochemical staining of the chest mass biopsy were consistent with metastatic hepatocellular carcinoma (HCC). The patient opted to pursue no further medical intervention and expired two weeks later. To the authors' knowledge, this is one of very few descriptions of isolated hepatocellular carcinoma found in the absence of a primary liver lesion and classical risk factors for hepatocarcinogenesis. This case highlights that HCC may present independently of liver lesions seen on imaging in a patient without clear signs or symptoms of liver. HCC should be considered in cases of isolated tumors with unclear primaries as ectopic carcinogenesis and occult primary malignancy are possibilities.

## 1. Introduction

Hepatocellular carcinoma (HCC) is one of the most common cancers worldwide, estimated to be responsible for over one million cancer deaths annually [1]. Important risk factors for HCC include cirrhosis secondary to either chronic hepatitis B or hepatitis C, obesity, diabetes, and related nonalcoholic fatty liver disease (NAFLD) [1]. It is uncommon for patients to present with HCC without the aforementioned risk factors.

The clinical presentations of HCC are highly variable. One case describes a patient presenting with oral pain and who was found to have HCC metastasized to the jaw [2]. However, even in unusual cases of HCC, it is exceedingly rare for this malignancy to present in the absence of a primary liver lesion [3, 4]. In this case, we present a patient with few overt risk factors for HCC who had an unusual presentation with no radiographic evidence of a primary tumor.

## 2. Case Presentation

An 82-year-old man with a past medical history of diabetes mellitus presented to the hospital with a two-week history of worsening shortness of breath, cough, and pain on the left anterolateral chest wall. Prior to this, the patient was only taking metformin for diabetes. He would visit his primary care physician yearly and had no previous lab abnormalities on routine examination. He was a former tobacco user but had no history of alcohol, or illicit substance use.

He was hemodynamically stable with an oxygen saturation of 90% on room air. Physical examination revealed decreased breath sounds of his left lower lung fields and exquisite tenderness to palpation of his left anterior chest wall with no crepitus. This area was mildly indurated.

**Figure 1:**
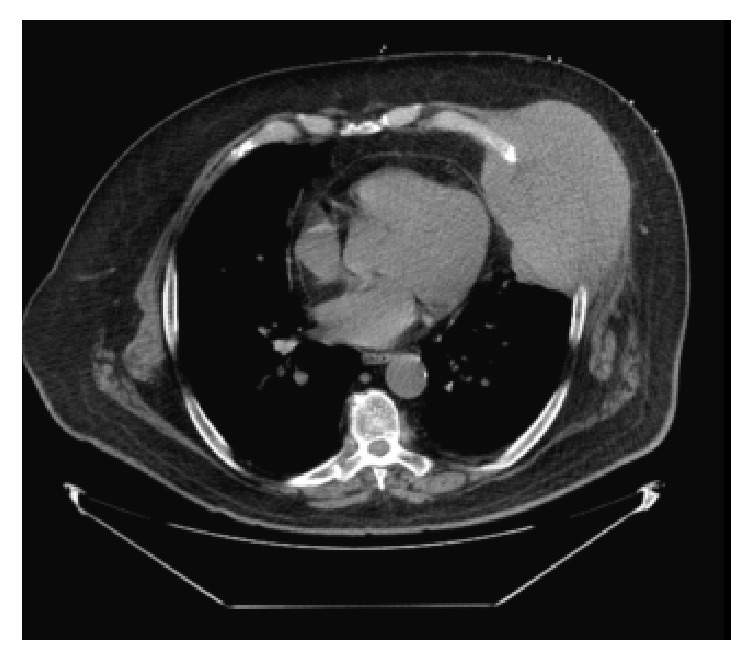
CT of chest with contrast showing 10 × 13 cm soft tissue mass in the left anterior lower hemithorax, from the left anterior 6th rib to the EO muscle and left pericardium, inseparable from LV myocardium.

**Figure 2:**
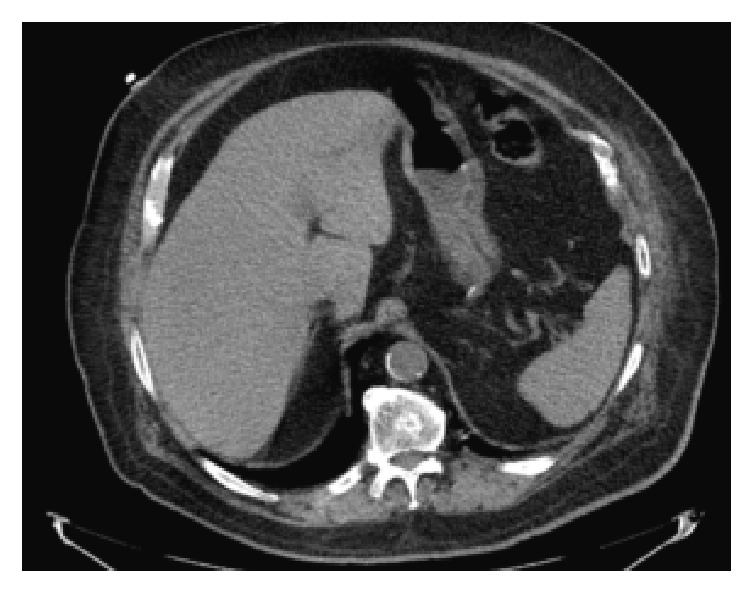
CT of abdomen with contrast showing unremarkable liver, spleen, pancreas, and adrenal glands.

Laboratory examinations showed a mild leukocytosis (11,700/*μ*L) with normal liver enzymes and basic metabolic panel. HIV and hepatitis screening panels were negative. A chest radiograph revealed a dense opacity at the periphery of the left lung base concerning for a mass. Further characterization through computed tomography (CT) of the abdomen and thorax with contrast demonstrated a 10 × 13.3 cm soft tissue mass in the left anterior lower hemithorax, extending from the left anterior sixth rib to the external oblique muscle and left pericardium (Figure 1). Furthermore, there were two exophytic masses in the left upper kidney and left lower kidney, highly suspicious for renal cell carcinoma. The visualized liver, spleen, pancreas, and adrenal glands were unremarkable with no masses (Figure 2). A biopsy of the chest wall mass with subsequent microscopic pathological examination was initially suggestive of metastatic renal cell carcinoma. However, immunohistochemical stains subsequently showed oncocytic cells with endothelial cell wrapping staining positive for HepPar-1 and Glypican-3, specific markers for HCC [5] and negative for CD10, CK7, and high molecular weight cytokeratin, markers of renal cell carcinoma [6, 7]. Additional immunostains showed tumor cells negative for PAX8 and a canalicular pattern with polyclonal CEA, supporting the final diagnosis of metastatic HCC in the chest wall without evidence of a primary liver source. After discussions with the patient and his family, the patient ultimately chose to not pursue further treatment or diagnostic testing for his malignancy. He opted for home hospice care given his belief in minimalist medicine.

## 3. Discussion

We have presented herein the unusual case of a gentleman with HCC presenting as a solitary lesion in the left anterolateral chest wall. On their own, chest wall tumors themselves are quite rare in the American and European populations [8].

Reports of HCC in the absence of a culprit primary liver lesion are exquisitely rare, especially as a first presentation of the disease [3, 4, 9]. HCC most commonly metastasizes to the lung, abdominal lymph nodes, and bone although extrahepatic metastasis typically occurs only in patients with advanced intrahepatic tumors [10]. Specifically, there are only a handful of cases with reported chest wall metastases of isolated HCC [3, 4, 11, 12]. The pathophysiology of such presentations is poorly understood. One purported mechanism is that the remote lesions stem from the spread of a microhepatocellular carcinoma [13]. In these scenarios, it is also possible that the primary hepatic tumor may simply have escaped delineation by established imaging modalities. Spontaneous regression of an original HCC lesion is another possibility, albeit unusual and exceedingly rare as well [14]. Malignant cells may spread from the primary site while the immune system brings about the resolution of the parent lesion.

An alternative possibility is that the malignant lesion arose from ectopic hepatocellular tissue. Ectopic liver is found infrequently and has been reported to be found in structures near the liver, omentum, retroperitoneum, and thorax [3]. Ectopic liver may be more prone to carcinogenesis than native tissue given that it does not possess a normally functioning vascular or ductal system [3]. Just like physiologically normal liver, ectopic liver tissue may be infected by viruses and develop steatosis or cirrhosis. In fact, Arakawa et al. reported that only 27% of reported patients with ectopic HCC possessed a normal mother liver [15]. Asselah et al. reported the case of a gentleman with chronic hepatitis C infection who presented with an indolent left chest wall mass ultimately found to be solitary extrahepatic HCC [3]. The patient did well after surgical resection of the mass, and no liver lesions were found by imaging even three years later. The presentation is reminiscent of our case; however, our patient did not test positive for any infections of the liver. Of note, ectopic HCC should be distinguished from metastasis, which carries a poor prognosis.

A final explanation is that the patient had developed NAFLD with an accompanying noncirrhotic liver. In this patient, the only salient risk factor for HCC was diabetes mellitus, which is known to predispose to the development of NAFLD. Furthermore, NAFLD itself may provoke hepatic carcinogenesis even in the absence of liver cirrhosis [16]. Among the United States population, NAFLD has a prevalence of roughly 30%, and the incidence of NAFLD-associated HCC has been notably increasing while the prevalence of nonalcoholic steatohepatitis (NASH) with advanced fibrosis is believed to be even higher at 66% [17].

Further work is needed to clinically and pathologically distinguish ectopic liver giving rise to HCC from isolated metastases of occult HCC lesions. It is possible that patients with ectopic HCC may have better long-term prognosis [3]. Such a distinction is critical as medical or surgical intervention is usually not offered in cases of distant tumor metastases. In cases of solitary HCC metastases with an unknown primary liver lesion, it may be reasonable to pursue more aggressive imaging of the liver and even biopsy in search of an occult liver lesion in order to make this delineation. Ectopic tissue has even been found from disparate organs and systems besides the liver, including the thyroid, pancreas, adrenal glands, thymus, and neurological system [18].

This case demonstrates that HCC may present independently of clear liver lesions on radiographic imaging, even in a patient without clear signs or symptoms of liver disease or multiple risk factors for malignancy. Although it is impossible to say which particular insult may lead to hepatocarcinogenesis, a systematic approach to diagnosing and treating risk factors—such as hepatitis, illicit substance abuse, and obesity—should be adopted. We propose that clinicians encountering the exceptional cases of isolated malignant lesions without detectable lesions in the native organ, such as the liver, attempt to distinguish ectopic carcinogenesis from occult primary lesions with metastases.
